# Impact of the presence and number of chromosomal abnormalities on the clinical outcome in Waldenström Macroglobulinemia: a monocentric experience

**DOI:** 10.1007/s00277-024-05770-4

**Published:** 2024-04-30

**Authors:** Nicolò Danesin, Laura Bonaldi, Annalisa Martines, Silvia Nalio, Roberta Bertorelle, Sofia Compagno, Raffaella Marcato, Sabrina Manni, Federico Scarmozzino, Marco Pizzi, Angelo Paolo Dei Tos, Alessandro Cellini, Greta Scapinello, Andrea Visentin, Livio Trentin, Francesco Piazza

**Affiliations:** 1 Hematology Unit, Department of Medicine, University of Padova, Via Giustiniani 2, 35128 Padua, Italy; 2 Immunology and Molecular Oncology Diagnostics, Veneto Institute of Oncology, IOV-IRCCS, Padua, Italy; 3 Veneto Institute of Molecular Medicine, Fondazione Per La Ricerca Biomedica Avanzata, Padua, Italy; 4 Surgical Pathology and Cytopathology Unit, Department of Medicine, University of Padova, Padua, Italy

**Keywords:** Waldenström Macroglobulinemia, Cytogenetics, Non-Hodgkin Lymphoma, Chromosomal abnormalities

## Abstract

The prognostic and predictive role of specific gene mutations in Waldenström Macroglobulinemia (WM) is well-ascertained whereas the clinical impact of chromosome aberrations is far less known. Recent work has provided initial evidence for an adverse prognostic impact of some aberrations, such as del(6q), while other studies suggest a possible relationship between some clinical features (*e.g.* advanced age and/or inflammatory status) and specific cytogenetic abnormalities. To add to the still limited knowledge on WM cytogenetics and its clinical implications, we herein report our experience in a cohort of WM patients across 23 years. Based on our retrospective study, we found that abnormal karyotype was more represented in older patients and maintained a statistically significant independence from other molecular, clinical, and biological features related to WM. The presence and number of cytogenetic aberrations correlated with inferior overall and progression-free survival outcomes regardless of the type of single chromosome aberration. Our data suggests that the role of the altered karyotype deserves to be further clarified especially in elderly WM patients, in whom cytogenetic abnormalities and disease biology appear to be characterized by a higher degree of complexity.

**Supplementary Information:**

The online version contains supplementary material available at 10.1007/s00277-024-05770-4.

## Introduction

Waldenström Macroglobulinemia (WM) is a rare indolent non-Hodgkin B cell lymphoma [1, 2]. Recently, clinical research has investigated whether the most frequent molecular alterations associated with the disease, i.e. *MYD88* and *CXCR4* gene mutations, may have a prognostic and/or a predictive role [3, 4]. Other studies have investigated the impact of the underlying cytogenetic alterations on survival outcomes, especially with regards to some aberrations such as deletion of long arm of the chromosome 6 (6q-), or deletion of the short arm of the chromosome 17 (17p-) [5]. A positive correlation between the presence of 6q- and the inflammatory form of WM has recently been described [6]. Furthermore, there seems to be a difference in terms of the number of chromosomal alterations between younger and very elderly WM patients [7].

Differently from other B-cell malignancies, the role of cytogenetics in WM has been investigated in fewer studies and only partially discussed in current guidelines [8, 9]. The work by the FILO group, one with the largest sample size reported, demonstrated the independent prognostic value of *TP53* abnormalities and high-complex karyotype (≥5 chromosome aberrations) in determining a shorter progression free survival (PFS) in WM [10].

In the present study, we retrospectively revised the cytogenetic data of WM patients collected between 2000 and 2023 in our institution, an academic center with experience on the treatment of this disease. The aim of the study was to assess potential correlations between cytogenetic aberrations and specific clinical and biochemical features (age, disease burden, inflammatory form, renal disorder). In addition, we tested the hypothesis that the numerosity of the cytogenetic alterations could have a prognostic role, therefore affecting survival or other time-to-event outcomes, independently from the impact of single, particular cytogenetic abnormalities.

## Material and methods

### Statistical analysis

Clinical records of WM patients followed between 2000–2023 at the Hematology Unit, University of Padova, Italy, have been used to collect data according to the protocol (4089/AO/17 and 3146/AO/24) approved by the Ethic Committee of the Azienda Ospedale-Università Padova.

Clinical, biochemical, molecular and cytogenetic findings obtained at diagnosis have been analyzed. Newly diagnosed patients were stratified as symptomatic or asymptomatic according to Consenus panel 1 of the 11th International Workshop on Waldenstrom Macroglobulinemia (IWWM) [11].

Continuous variables were described by median (interquartile range, IQR) while categorical ones were described by frequency (percentage). Continuous variables were compared with the Mann–Whitney U test, categorical ones were compared with the Chi-square test or the Fisher’s exact test, as appropriate. Major response rate was defined as the frequency of partial, very good and complete response achieved after first line therapy. Survival outcomes considered were overall survival (OS) and progression free survival (PFS). OS was defined as the time from diagnosis to death from any cause while PFS as the time from diagnosis to progression or death from any cause.

TTI (Time to initial treatment) and TTNT (Time to next treatment) outcomes were also explored for asymptomatic and symptomatic WM patients, respectively.

TTI was defined as the time from diagnosis to the start of a first line therapy whereas TTNT was defined as the time from the start of a first line to the start of the second line treatment.

The impact of variables on survival outcomes (OS, PFS) was investigated with the Cox proportional hazard regression model, by univariate analysis in different subgroups. Results were reported as hazard ratios (HR) and 95% confidence intervals. Missing data were accurately excluded from the analysis. Survival curves were built with the Kaplan–Meier method and compared by the log-rank test. Statistical significance was considered for p-value < 0.05. Statistical analysis was performed on RStudio version 2022.07.2.

### Chromosome banding and molecular analyses

Chromosome banding analysis (CBA) was performed on bone marrow aspirates after a 72 h stimulation with 500 µM CpG ODN DSP30 mitogen (Roche, Risch, CH) + 20 U/mL interleukin-2 (IL-2) (Roche) or unstimulated culture, when circulating tumor cells were present) [12]. After overnight exposure with 1 µg of KaryoMax® Colcemid® Solution (Life technologies Corporation, Grand Island, NY, USA), cells were harvested by adding 0.075 M KCL hypotonic solution and incubated for 30’ at room temperature, followed by three times fixation with Carnoy’s solution. Wright’s stain was diluted in Söerensen’s Buffer (0.06 M/l Na2HPO4/0.06 M/l KH2PO4) for G-Banding analysis. The slides were analyzed using the Metafer automated acquisition system (MetaSystems s.r.l., Milan, Italy), and the karyotype was described after the analysis of at least 25 metaphases in accordance with international guidelines (ISCN2020) [13].

Complex karyotype (CK) and high complex karyotype were defined respectively as the presence of 3 or more and 5 or more clonal cytogenetic abnormalities.

To detect *MYD88* L265P mutation on bone marrow aspirates, a highly sensitive allele-specific PCR (AS-PCR) was used. Two non-competitive PCRs were run to identify the wild-type and mutated sequences by using 2 forward primers, one specific for the wild-type allele and the second one specific for the mutated [14].

To investigate *CXCR4* mutations on bone marrow aspirate we used 2 allele-specific PCR, one for the c.1013C > G mutation and another for the c.1013C > A and we performed sequence analysis of exon 2 to identify other non-sense or frameshift mutations, even though with a lower sensitivity [15, 16].

## Results

A total number of 85 out of 207 newly diagnosed WM patients was successfully karyotyped by chromosome banding between 2000 and 2023. The median follow-up period for the entire cohort was 51 (19–81) months.

The cohort of patients that was studied comprised 64 (75.3%) out of the initial 85, due to the loss of 13 patients during follow up, lack of data in 6 cases, and the exclusion of other 2 patients because of cytogenetic analysis being performed after the diagnosis (Table 1).

**Table 1:** Baseline characteristics of WM patients with normal and abnormal karyotype

	Normal karyotype n = 34	Abnormal karyotype n = 30	*P* value
Age, years, median (IQR)	65 (58–70)	72 (66–82)	**0.003**
CIRS > 6, n (%)	15/27 (0.56)	16/28 (0.57)	1.00
ECOG PS ≥ 2, n (%)	5/28 (0.18)	2/26 (0.08)	0.42
Sympomatic patients, n (%)	12/34 (0.35)	13/30 (0.43)	0.57
Hb ≤ 115 g/L, n (%)	13/34 (0.38)	10/30 (0.30)	0.68
PLTs ≤ 100 × 10^9^/L, n (%)	4/34 (0.12)	0/30 (0.00)	0.11
β2-microglobulin > 3 mg/L, n (%)	14/19 (0.74)	10/13 (0.77)	0.84
MC IgM, g/L, median (IQR)	12.00 (6.90–20.39)	14.65 (8.51–25.75)	0.24
CRP, mg/L, median (IQR)	4.80 (2.90–35.00)	5.37 (2.90–10.00)	0.80
BM infiltration, %, median (IQR)	55 (19–76)	77 (65–98)	0.79
Creatinine, micromol/L, median (IQR)	75 (63–88)	77 (65–98)	0.79
MYD88^L265P^, n (%)	26/31 (0.84)	24/27 (0.89)	0.58
CXCR4 mut, n (%)	5/17 (0.29)	2/15 (0.13)	0.40
IPSSWM			
Low, n (%) Intermediate, n (%) High, n (%)	6/12 (0.50) 3/12 (0.25) 3/12 (0.25)	3/13 (0.23) 3/13 (0.23) 7/13 (0.54)	0.14
Second cancer, n (%)	3/34 (0.08)	10/30 (0.30)	**0.03**
Need of therapy, n (%)	19/34 (0.55)	25/30 (0.83)	**0.02**
Major response rate, n (%)	16/26 (0.62)	7/18 (0.39)	0.13
2nd or more treatment lines, n (%)	8/34 (0.24)	9/30 (0.30)	0.56

*BM*: Bone marrow; *CIRS*: Cumulative Illness Rating Scale; *CRP*: C-reactive protein; *ECOG-PS*: Eastern Cooperative Oncology Group Performance Status; *Hb*: Hemoglobin; *IPSSWM*: International Prognostic Scoring System on Waldenström Macroglobulinemia; *IQR*: Interquartile range; *MC*: Monoclonal Component

Overall, 67% (43/64) of cases were studied after mitogen stimulation with CpG oligonucleotide + IL2. Eighty-three% (25/30) and 53% (18/34) of cases, respectively, for the WM patients with altered karyotype and for those with normal karyotype, were studied with this procedure.

At diagnosis 25 out of 64 patients (39%) were identified as symptomatic and they were equally distributed between the normal and altered karyotype subgroup. In terms of CBA, 30 out of 64 patients (46.9%) showed an abnormal karyotype with a single chromosome change in 17/30 (56.6%) cases, two abnormalities in 7/30 (23.3%) patients and a CK in 6/30 (20%) patients. Only 2 patients exhibited a karyotype with more than 5 clonal chromosomal aberrations (high-CK). Considering the type of aberrations, structural abnormalities were detected in 13/30 (43.3%) cases followed by numerical changes in 11/30 (36.6%). Both structural and numerical aberrations were found in a minority of cases, specifically in 6 of 30 (20.0%).

In line with other previously published studies, the most frequent aberration was the deletion of the long arm of chromosome 6 (6q-) in 7/30 (23.3%) patients, followed by the trisomy of the chromosome 3 (+ 3) in 7/30 (23.3%) patients and the deletion of long arm of the chromosome 11 (11q-) detected in 4/30 (13.3%) cases. The loss of Y chromosome (-Y) similarly to the trisomy of the chromosome 18 (+ 18) and to the trisomy of the chromosome 12 (+ 12) was described in 4/30 (13.3%) patients for each reported aberration (Table 2).

**Table 2:** Summary of cytogenetic analysis

Cytogenetic feature	Frequency (%)
Karyotype	
Abnormal	30/64 (46.9%)
Normal	34/60 (56.6%)
Number of aberrations in the karyotype	
1	17/30 (56.6%)
2	7/30 (23.3%)
≥ 3 (complex and high-complex)	6/30 (20.0%)
Type of aberrations in the karyotype	
Numerical only	11/30 (36.6%)
Structural only	13/30 (43.3%)
Numerical and structural	6/30 (20.0%)
Recurrent aberrations in the karyotype (in more than 3 cases)*	
Deletion 6q	7/30 (23.3%)
Trisomy 3/partial + 3	7/30 (23.3%)
Deletion 11q	4/30 (13.3%)
Trisomy 12/partial + 12	4/30 (13.3%)
Trisomy 18	4/30 (13.3%)
Loss of Y	4/30 (13.3%)

*No Trisomy 4 was isolated

Detailed karyotypes for each patient are reported in Supplementary material (Table S2).

In the abnormal karyotype subgroup, we observed a higher prevalence of secondary cancers (30.00% vs 8.82%, *p *= 0.03), as we recorded 10 patients diagnosed with solid tumors (cystic pancreatic cancer, bowel cancer, prostate cancer, two with kidney cancer, bladder cancer, papillary thyroid cancer, three with skin cancer). At variance, in the normal karyotype subgroup, only three patients developed solid tumors (liver cancer, esophageal cancer, salivary glands cancer). No secondary myelodysplastic syndromes or myeloid tumors were observed.

When comparing WM patients with and without cytogenetic abnormalities, no significant correlation was observed between the presence of cytogenetic abnormalities and disease burden, renal impairment, inflammatory phenotype, comorbidity scores, IPSSWM and molecular features. However, we found that WM patients with at least one abnormal clone were older at diagnosis (median age 72 years *vs* 65 years, *p *= 0.003) (Table 1).

Moreover, advanced age was also found to correlate with the number of abnormalities, since WM patients with CK had a median age significantly higher than that of the subjects with 2 or less cytogenetic aberrations (85 *vs* 66 years, respectively, *p* < 0.001). Similarly, patients with 2 or more cytogenetic aberrations were older than patients with 0 or 1 alterations (median age 80 vs 66 years, respectively, *p *< 0.001) (Table 3).

**Table 3:** Median age at diagnosis according to the karyotype

Subgroups	Median age at diagnosis, years (IQR)	*P *value
Abnormal karyotype	72 (66–82)	0.003
Normal karyotype	65 (58–70)	
≥ 2 cytogenetic aberrations	80 (72–84)	< 0.001
< 2 cytogenetic aberrations	66 (59–72)	
Complex karyotype	85 (83–87)	< 0.001
No complex karyotype	66 (59–73)	

*IQR* = interquartile range

Among patients with a normal karyotype, 19 out of 34 (55%) required treatment whereas among those with an altered karyotype 25 out of 30 (83%) needed therapy with significant difference (*p* = 0.02). The criteria for initiating first-line therapy were evenly distributed between the two groups. Anemia, hyperviscosity, and neuropathy were the most prevalent causes to dictate a treatment in our population (Table 1).

The number of subsequent lines of therapy administered after the first one was also similar between the two groups. Conventional chemoimmunotherapy was the most frequent type of treatment administered. Bruton Tyrosine Kinase (BTK) inhibitors were used at similar frequency in the two groups and a major response after the first line therapy was achieved in 4 out of 5 cases.

Major response rate to first line therapy tended to be higher in the normal karyotype subgroup although statistical significance was not reached (63% vs 38%, *p* = 0.12). No substantial differences were seen in disease progression rate after first line therapy (20% vs 23.5%) during the entire follow up period. The percentage of death events was superior in patients with abnormal cytogenetics when compared to those with a normal karyotype (8/30 [27%] and 4/34 [12%], respectively). Of the patients for whom the cause of death could be recognized, two died due to secondary cancers likely not related to WM-associated treatments (esophageal and hepatocellular carcinoma) in the abnormal karyotype subgroup, while in the normal karyotype subgroup three patients died due to sepsis and one due to WM transformation to an aggressive lymphoma.

**Fig. 1:**
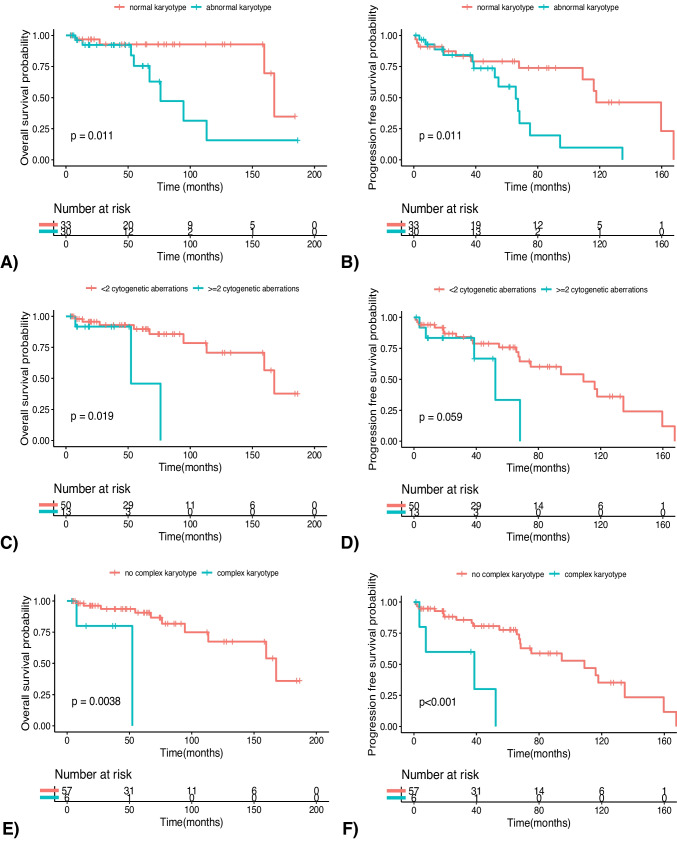
Overall survival (OS) and progression free survival (PFS) for: **(1A, 1B)** WM patients with abnormal (green) *versus* normal karyotype (red); **(1C, 1D)** WM patients with ≥ 2 cytogenetic aberrations (green) *versus* < 2 cytogenetic aberrations (red); **(1E, 1F)** WM patients with complex karyotype (green) *versus* patients without complex karyotype subgroups (red)

When performing survival analyses, we found that WM patients with cytogenetic aberrations displayed inferior median overall survival (mOS) compared to those with a normal karyotype (76.1 vs 167.7 months, respectively [p value = 0.01]) and a similar trend was noted for the median progression free survival (mPFS) in the two subgroups (65.8 vs 117.8 months, respectively [p value = 0.01]) (Fig. 1A and B). Furthermore, the PFS curves diverged even more after 40 months. Additionally, no clinical and biochemical features related to WM disease were distributed differently between the groups beyond this time point.

WM patients with ≥ 2 chromosome changes exhibited an inferior mOS compared to those with 1 or 0 abnormalities (52.3 *vs* 167.7 months, respectively [*p* value = 0.02]) and a trend towards an inferior mPFS was also observed (52.3 vs 108.9 months, respectively [*p* value = 0.06]). The same held true for cases with or without CK. In fact, the CK subgroup exhibited significantly shorter mOS (52.3 vs 167.7 months respectively [p value = 0.004]) and mPFS (38.6 vs 108.9 months, respectively [*p *value < 0.001]) (Table 4).

**Table 4:** Overall and progression free survival outcomes according to the karyotype

Subgroups	Median OS (months)	HR (CI 95%)	P value	Median PFS (months)	HR (CI 95%)	P value
Normal karyotype	76.1	4.35 (1.27–14.86)	0.01	65.8	2.90 (1.24–6.83)	0.01
Abnormal karyotype	167.7			117.8		
≥ 2 cytogenetic aberrations	52.3	5.28 (1.15–24.31)	0.02	52.3	2.69 (0.92–7.81)	0.06
< 2 cytogenetic aberrations	167.7			108.9		
Complex karyotype	52.3	9.14 (1.56–26.11)	0.004	38.6	6.05 (1.85–19.79)	< 0.001
No complex karyotype	167.7			108.9		

*HR* = Hazard Ratio; *CI* 95% = 95% confidence interval; *OS* = Overall Survival; *PFS* = Progression Free Survival

As mentioned above, the described difference in terms of survival outcomes was maintained even with the increase of the number of aberrations (Fig. 1C-F). A univariate Cox proportional hazard regression analysis was conducted, examining various features. None of the considered variables (disease burden, renal impairment, inflammatory phenotype, comorbidity scores, and molecular features) were found to have a significant correlation with the observed differences in survival outcomes, thus suggesting an independent role of cytogenetic abnormalities in determining inferior OS and PFS outcomes in WM patients.

Furthermore, specific outcomes for asymptomatic (Time to initial treatment, TTI) and symptomatic (Time to next treatment) WM patients were also investigated.

We found a different TTI between the abnormal and normal karyotype subgroups in the symptomatic condition (47.0 vs 225.0 months respectively [*p* value = 0.01]. (Supplementary Figure S1, Table S1). No significant differences were observed in TTNT for the symptomatic patients.

## Discussion

In the present study, we have analyzed the features and the outcomes of a cohort of WM patients from an academic institution, dividing the included subjects based on the presence and the number of chromosomal abnormalities detected at diagnosis.

In line with the above-mentioned French study, we detected a similar prevalence of the deletion of 6q (23.3%) [10]. However, our findings also revealed a high frequency of trisomy of the chromosome 3 (23.3%) which was among the less reported alterations in the French investigation. This chromosomal aberration is more frequent in marginal zone lymphomas than in WM [17]. However, of 7 WM patients characterized by trisomy 3, only one was MYD88L265P wild type while the others were MYD88L265P mutated, a hallmark which is more typical for WM [3].

Higher frequency of Y loss (13.3%) and 11q deletion (13.3%) were also detected. Notably, trisomy of chromosome 4 did not emerge as a recurrent alteration in our case series, probably due to the small size of our cohort [10]. The search for chromosome 17 deletion has been performed in a minority of cases and for this reason the frequency of this alteration could not be compared to that described in previously published studies. The prevalence of complex karyotype showed similar frequency (13.3%) to that described in the FILO study, even if we reported a lower number of patients with High-CK [10]. With the exception of 6q-, which is typical of WM, all the reported alterations are recurrent in low-grade B-cell lymphoproliferative disorders, and the varying frequency of each is likely dependent on the size of the studied sample and the stage of the disease under investigation.

To the best of our knowledge, no previously published studies have investigated the potential association between age and cytogenetic abnormalities. Indeed, our analysis suggests a correlation between older age and a higher prevalence of cytogenetic aberrations. This association appeared to be stronger when comparing patients with CK to patients without CK, confirming our recently published findings in a study involving very elderly WM patients [7]. A longstanding condition of monoclonal gammopathy preceding the diagnosis of Waldenström Macroglobulinemia and the treatment could probably influence karyotype characteristics determining inferior survival outcomes. Older age could also be responsible for the observed higher rate of secondary malignancies in the aberrant cytogenetic subgroup, which seem to be unrelated to WM-related therapy exposure in view of the fact that no myeloid disorders have been recorded. However, neither second tumors nor lymphoma were prevalent causes of death in our series.

Unlike the aforementioned studies, we did not find a positive correlation between disease burden or inflammatory WM and cytogenetic abnormalities. Nonetheless, elevated IPSSWM scores were found in the abnormal karyotype subgroup, but this is possibly attributable to a major prevalence of older patients. When evaluating the single cytogenetic aberrations (either structural or numeric) we did not find an impact on survival outcomes. These results, however, could be biased by the small numerosity of the cohort. In all the comparisons among subgroups (normal *versus* abnormal karyotype, ≥ 2 *versus* < 2 cytogenetic aberrations, CK *versus* normal karyotype) the independence of cytogenetics from clinical or biological variables was maintained, with the significant exception for age at diagnosis. 

On the other hand, in comparing the subset of patients with altered karyotype to those without chromosome changes, we found a trend towards a correlation between inferior OS and PFS and the increase of the number of cytogenetic aberrations (Fig. 1). Within the first 40 months following diagnosis, patients with an altered karyotype exhibited a comparable rate of relapse events to those with a normal karyotype (Fig. 1B). Beyond this time point, the frequency of relapse events substantially increased for the individuals with altered cytogenetics. While the identification of the underlying cause(s) of this late divergence requires further investigation, it is conceivable that the presence of chromosomal abnormalities may confer increased inherent resistance to subsequent line of therapies thereby leading to more relapses or death events. Additionally, no clinical and biochemical difference were noticed between groups beyond this time point.

Differently from what we have reported here, a recent Chinese study did not find statistically significant survival differences in WM patients with two or more cytogenetic aberrations compared to patients with a single alteration or a normal karyotype [18]. However, in line with previously published data, we found no substantial differences in terms of response to therapy, nor we found any specific relationship between cytogenetics and the type of therapy administered [10, 19]. Moreover, even if with the limit of a small numerosity, it seems that the use of BTKi might be associated with better response rates when employed in elderly patients with altered karyotype, a finding that should deserve further investigation. Furthermore, we have not found specific causes determining the inferior OS in the aberrant cytogenetic subgroups as the spectrum of causes of death in our patients was heterogeneous.

Likely, the small numerosity of the cohort, as well as the low number of TTI and TTNT events for asymptomatic and symptomatic patients, respectively, did not allow to detect significant differences from this analysis. Nevertheless, the data suggest that asymptomatic WM patients with abnormal karyotype could display a shorter median TTI than asymptomatic WM patients with normal karyotype (47,1 vs 225,16 months, respectively, *p* = 0,01).

To note, our cohort demonstrated to display features comparable to those of larger cohorts in retrospective studies such as the French one in terms of karyotype alterations, with the exception for the absence of consistent *TP53* mut/del. While our study is limited by the relatively small numerosity and the retrospective design, it has the advantage that the cytogenetic analysis was handled by a single laboratory. However, a drawback is represented by the large spectrum of different 1st line treatment regimens employed during the long follow-up period (23 years) of this study which could partially modify the interpretation of PFS and OS results.

In conclusion, our data suggest an independent role of the karyotype on survival outcomes in WM patients. The impact on outcomes seems to be proportional to the number of abnormalities, being increasingly worst for patients with one, two, three or more cytogenetic aberrations. With the exception for older age at diagnosis, no positive correlation between altered karyotype and disease burden, *MYD88*/*CXCR4* mutation or inflammatory WM has been found. The positive correlation between age and the number of cytogenetic aberrations could at least in part be related to a different biological background in this group of patients. Considering the heterogeneity of cytogenetic aberration in WM and the absence of a real disease-qualifying aberration, we thought it would be clinically meaningful to investigate the impact on survival outcomes of the number of chromosomal aberrations instead of that of the single cytogenetic abnormalities. Therefore, our study underlines the importance also of karyotype testing at diagnosis in WM patients, to obtain better outcome predictions in this rare lymphoid malignancy.

### Supplementary Information

Below is the link to the electronic supplementary material.

Supplementary file1 (DOCX 60 KB)

## Data Availability

The data that support the findings of this study are available upon request from the corresponding author.
