# Supplementary material for: Impact of the presence and number of chromosomal abnormalities on the clinical outcome in Waldenström Macroglobulinemia: a monocentric experience
Source: Ann Hematol. 2024 Apr 30;103(8):2961–8. doi: 10.1007/s00277-024-05770-4 (PMC11283403; doi:10.1007/s00277-024-05770-4)
Supplement: Supplementary file 1 — Supplementary file1 (DOCX 60 KB) [file 277_2024_5770_MOESM1_ESM.docx]

**1AS)****1BS)**

**1CS)****1DS)** **1ES)**

**FIGURE 1S. Time to initial treatment (TTI) and Time to next treatment (TTNT) for: (1AS, 1BS)** WM patients with abnormal (green) *versus* normal karyotype (red); **(1CS, 1CS)** WM patients with ≥2 cytogenetic aberrations (green) *versus* <2 cytogenetic aberrations (red); **(1ES)** WM patients with complex karyotype (green) *versus* patients without complex karyotype subgroups (red).

**TABLE 1S.** **Time to initial treatment (TTI) and Time to next treatment (TTNT) respectively for asymptomatic and symptomatic WM patients according to the karyotype.**

| **Subgroups** | **Median TTI (months)** | **HR (CI 95%)** | **P value** | **Median TTNT**  **(months)** | **HR (CI 95%)** | **P value** |
| --- | --- | --- | --- | --- | --- | --- |
|  | | | | | | |
| Normal karyotype | 225.0 | 1.07 (0.46-2.44) | 0.01 | NR | 1.19 (0.16-8.63) | 0.86 |
| Abnormal karyotype | 47.0 |  |  | 153.5 |  |  |
|  | | | | | | |
| ≥ 2 cytogenetic aberrations | 15.6 | 1.30 (0.33-1.83) | 0.36 | 136.5 | 1.26 (0.11-13.93) | 0.85 |
| < 2 cytogenetic aberrations | 160 |  |  | NR |  |  |
|  | | | | | | |
| Complex karyotype | - | - | - | 136.5 | 2.03 (0.18-22.56) | 0.56 |
| No complex karyotype | - |  |  | NR |  |  |

TTI = Time to initial treatment; TTNT = time to next treatment; HR = Hazard Ratio; CI 95% = 95% confidence interval; NR = not reached;

**TABLE 2S. Abnormal karyotypes of the entire cohort and number of clonal abnormalities**

| **Patient** | **Karyotype** | **Number of**  **cytogenetic aberrations*** |
| --- | --- | --- |
| 1 | 46,XY,del(6)(q12)[3]/46,XY[23] | 1 |
| 2 | 45,X,-Y[13]/46,XY[12] | 1 |
| 3 | 47,XX,+8[4]/46,XX[23] | 1 |
| 4 | 47,XX,+3[2]/46,XX[23] | 1 |
| 5 | 46,XY,del(11)(q14)[11]/46,XY[14] | 1 |
| 6 | 47,XY,+3[18]/48,idem,+18[2]/46,XY[6] | 2 |
| 7 | 47,XY,+12[3]/46,XY[22] | 1 |
| 8 | 48,XY,+3,+18[7]/46,XY[18] | 2 |
| 9 | 45-46,XY,del(6)(q21),inc[cp9]/46,XY[21] | 1 |
| 10 | 48,XY,+3,+12,t(14;18)(q32;q21)[16]/46,XY[9] | 3 |
| 11 | 46,XX,t(12;13;16;17)(q13;q34;q12;q21)[11]/46,XX[19] | 1 |
| 12 | 45,X,-X[8]/46,XX[17] | 1 |
| 13 | 46,XY,del(11)(q21q23)[7]/46,XX[5] | 1 |
| 14 | 43,XY,add(6)(q13),inv(9)(p21q22),add(14)q32),-15,-20,-21[4]/43,idem,-13,der(18)t(3;18)(q21;q21),+21[13]/44,idem,+7[2]/46,XY[6] | ≥ 3 |
| 15 | 49,XX,+3,+12,+18[2]/46,XX[119] | 3 |
| 16 | 46,XX,del(11)(p11.2)[6]/45~46,idem,t(X;6)(q25-27;q23),del(8)(p21),der(8;17)(q10;q10)[cp12]/46,XX[9] | ≥ 3 |
| 17 | 46,XX,t(11;21;18)(q21;p13;q21)[24]/46,XY[1] | 1 |
| 18 | 46,XX,add(10)(q26)[11]/46,XX[14] | 1 |
| 19 | 46,XX,del(6)(q21)[5]/46,idem,del(16)(q21-23)[3]/46,XX[17] | 2 |
| 20 | 47,XX,+3[8]/46,XX[17] | 1 |
| 21 | 46,XX,i(6)(p10)[cp2]/46,XX[23] | 1 |
| 22 | 47,XY,+18[3]/46,XY[24] | 1 |
| 23 | 46,XY,del(13)(q14q22)[11]/46,idem,t(7;10)(q32;q24)[14] | 2 |
| 24 | 46,XY,del(6)(q21),del(7)(q21)[23]/46,XY[2] | 2 |
| 25 | 46,XY,del(11)(q14q24)[15]/46,XY[10] | 1 |
| 26 | 47,XY,t(1;13)(q42;q14),-13,+add(19)(q13)[25] | 3 |
| 27 | 45,X,-Y,del(6)(q14)[6]/46,XY[19] | 2 |
| 28 | 45,X,-Y[17]/46,XY[8] | 1 |
| 29 | 47~48,XY,+12,+19,der(19)t(4;19)(q21;p13)t(4;?)(q3?;?)[cp24]/46,XY[1] | ≥ 3 |
| 30 | 45,X,-Y,del(6)(q24),inc[9]/46,XY[9] | 2 |

*The number of aberrations was counted according to ISCN 2020; ≥3 aberrations correspond to a complex karyotype
